# Prevalence and ethnic/racial disparities in the distribution of pediatric injuries in South Florida: implications for the development of community prevention programs

**DOI:** 10.1186/s40621-017-0108-9

**Published:** 2017-04-17

**Authors:** Carmen Ramos Irizarry, Patrick C. Hardigan, Mark G. Mc Kenney, Gretchen Holmes, Rudy Flores, Brenda Benson, Ascension M. Torres

**Affiliations:** 1 Department of Surgery, Kendall Regional Medical Center, 11750 SW 40th St, Suite 701, Miami, FL 33175 USA; 2 Biostatistics Research Division, Health Professions, Nova Southeastern University, Ft Lauderdale, FL USA; 3 Research Division, Kendall Regional Medical Center, Miami, FL USA

**Keywords:** Prevalence, Trauma, Race, Ethnicity, Pediatric, Disparities, Geographic mapping, Prevention programs, Precede framework

## Abstract

**Background:**

The state of Florida continues to report significant gender, ethnic and racial disparities in trauma incidence, access to care and outcomes in the adult population. Our objective was to assess pediatric injury profiles and ethnic/racial disparities of specific injuries in a Regional Trauma Center (TC) in South Florida.

**Methods:**

Retrospective data from November 2011 to December 2015 were obtained from the Level 2 TC registry for children ≤21 years old. Demographic, injury pattern, geographic area, injury scores and treatment data were analyzed.

**Results:**

One thousand six hundred ten patients, ages 0–21 years were cared for at the TC from 2011 to 2015.73% were males. Mean age = 15.7 years. Mortality was 2.3%. Using zip code data and using geographic mapping, we identified two main clusters where injuries were occurring. A multinomial regression analysis demonstrated that Hispanics had higher risks of falls (RR 10.4, 95% CI 2.7–29), motorcycle accidents (RR 3.7, 95% CI 1.7–8.2) and motor vehicle accidents (RR 6.4, 95% CI 3.6–11.4). Black/African American children had higher risks of gunshot wounds and resultant mortality (*p* < 0.01).

**Conclusion:**

There were racial, ethnic and gender disparities in the patterns of injury and outcomes among the youth attended at our TC. Geographic mapping allowed us the identification of the zones in South Florida where injuries were occurring. Understanding the differences and using geographic mapping to identify regions of higher prevalence will complement planning for prevention programs.

## Background

Injury, although considered a preventable public health problem, is the leading cause of death in Florida residents’ ages 1–44 years (Florida Department of Health, 2016). Injury as a result of trauma affects all ages, races, ethnicities and socioeconomic groups; however, significant disparities exist in its incidence and outcomes (Unintentional injury death; Brown 2010; Florida Department of Health 2016; Gilchrist and Parker 2014; Haider et al. 2008; Huang, et al. 2016). In 2015, Florida’s age-adjusted injury rates were higher than the national average, with significant racial and ethnic disparities observed in the injury patterns and rates (Unintentional injury death.). Non-Hispanic adult residents have higher rates of both fatal injuries and non-fatal hospitalizations than Hispanic residents (Florida Department of Health 2016). White adults have the highest rates of fatal injuries, followed by Blacks and Other (Florida Department of Health 2016). For children ages 1–21 years, injury crude death rates reported for 2013–2015 period were 13.4/100,000 with significant gender, racial and ethnical disparities observed throughout the State (Unintentional injury death.). Incorporating injury prevention programs in the community has the potential of reducing disparities and inequities in the care of injured children.

The Safe Kids Florida injury prevention coalitions have influenced the reduction of unintentional injury fatality rates in participating counties by 28%; however, there are still multiple counties without Safe Kids coalitions or sustainable injury prevention programs (Florida Department of Health 2016).

The first step in the creation of injury prevention programs requires the identification of the population at risk. The purpose of this study was to determine the prevalence and ethnic/racial patterns of injury in the pediatric population attended at a Level 2 Regional Trauma Center. Our intention was to assess injury profiles and to determine the presence of ethnic/racial disparities associated to specific types of injuries that could help us intensify and develop injury prevention programs in our community.

## Methods

The Institutional Review Board granted exemption for this retrospective analysis. Data from November 1, 2011 to December 31, 2015 were obtained from the Level 2 Trauma Center (TC) registry, (currently a provisional Level 1) at a Regional Medical Center in South Florida for children ≤ 21 years old. All data were de-identified for statistical analysis.

Demographic, injury patterns, geographic area, injury scores and treatment data were analyzed. Descriptive statistics were calculated for all study variables. This included mean and standard deviation for continuous measures and counts and percentages for categorical variables. A logistic regression model using a penalized likelihood ratio test was performed to ascertain the effects of age, race/ethnicity, Injury Severity Score (ISS) (Copes et al. 1988), Trauma Related Injury Severity Scores (TRISS) (Boyd et al. 1987), Intensive Care Unit (ICU) days, and hospital length of stay (HLOS) on the likelihood of patient survival. A multinomial regression model was used to ascertain the effects of race/ethnicity on the likelihood of suffering specific injuries classified as gunshot wounds, stabbing, motor vehicle crashes, motorcycle crashes, pedestrian injuries, and other injuries.

We used a K-means cluster analysis of both longitude and latitude to model where the injured person lived. K-means cluster analysis uses an algorithm that minimizes the within sum of squares to identify clusters of data points. To gain a better understanding between injury type and location in Miami-Dade County, we also created a GIS map that plots injury type by location. For a visual representation, the clusters were plotted on a map of Florida using the software ggplot21 (Wickham 2009). All statistical analyses were conducted using R version 3.3.1.2 (R Core Team 2016). A value of *p* < 0.05 was used for statistical significance.

## Results

There were 1610 patients with ages ranging from 0 to 21 years cared for at the Regional TC from 2011 to 2015. Seventy-three percent were males. Mean age was 15.7 years. Approximately 74% of the population was in the 16–21 years’ category, 6.2% in 11–15 years, 4.1% in 6–10 years, and 14% in 0–5 years. Sixty- six percent were White, 2.5% Other, Unknown, or Asian/Native American and 31% were Black/AA. Forty-eight percent were Hispanic/Latino. Mortality rates were 2.3%, all exclusively observed in males in the 16–21 years’ group. Table 1 describes the demographic and clinical characteristics. Median initial GCS was 14, median ISS = 7 (range 1–75), and TRISS scores were 0.96 (range 0.001–0.999)). The mean ICU stay was 1.62 days (range 0–46 days). Maximum hospital stay was 95 days, with a mean of 3.94 days (SD 6.13).

**Table 1:** Demographic and Clinical characteristics

	N	Mean	SD	Min	Max
Age (yrs)	1610	15.7	6.3	0	21
Race	1610	%			
American Indian		0.5			
Asian		0.9			
Black/AA		31.0			
White		66.5			
Other		0.5			
Unknown		0.6			
Ethnicity	1610	%			
Hispanic		48.0			
Not-Hispanic		51.4			
Unknown		0.6			
	N	Mean	SD	Min	Max
ICU LOS (days)	1609	1.72	4.07	1	46
Hospital LOS (days)	1610	3.94	6.13	1	95
	N	Median		Min	Max
GCS	1588	14		3	15
ISS	1608	7		1	75
TRISS	1252	0.96		0	1.0

**Fig. 1:**
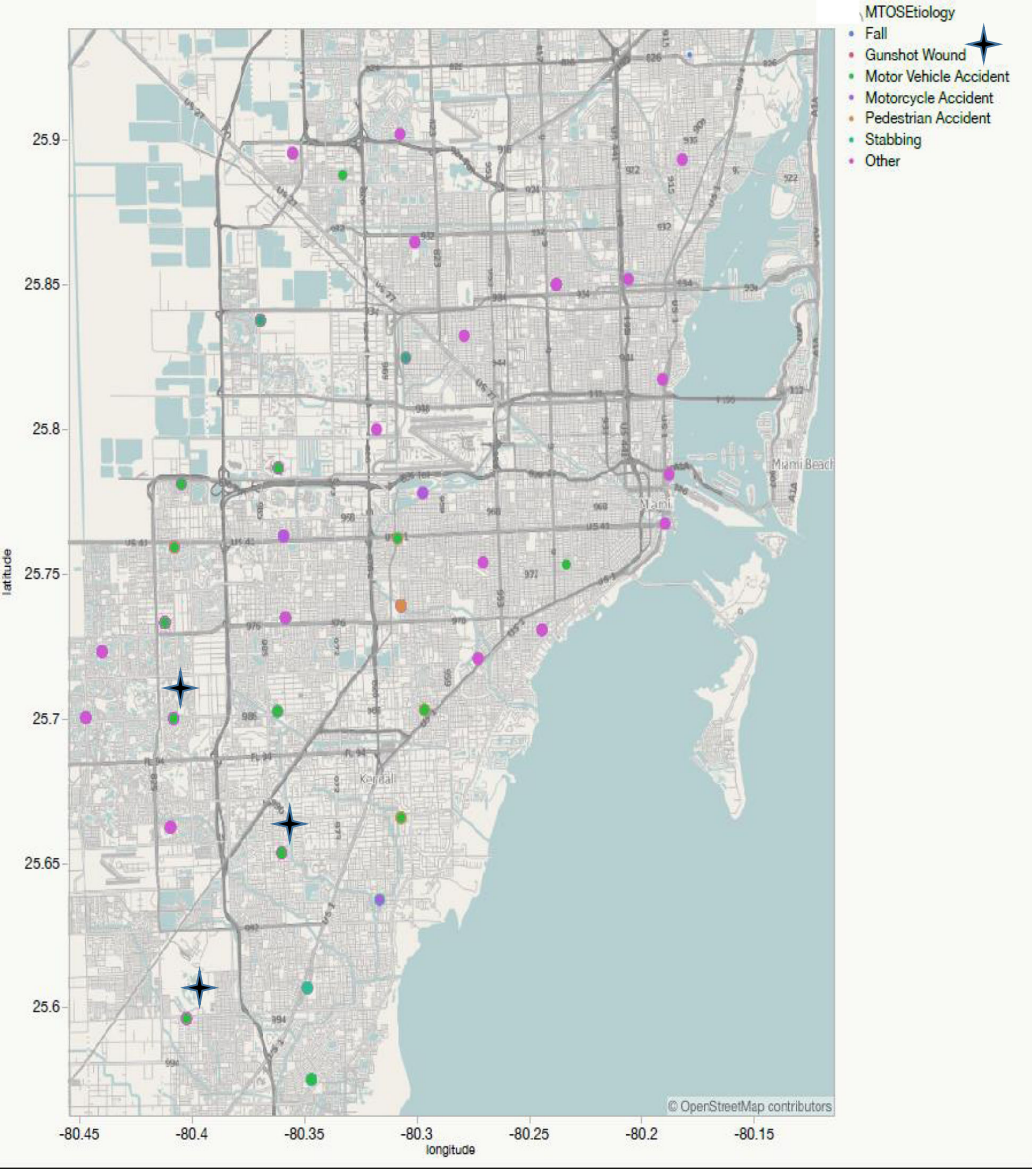
Distribution of injuries by zip code in Miami-Dade County. Fatalities due to gunshot wound injuries occurred within the same residential zip code zone

**Fig. 2:**
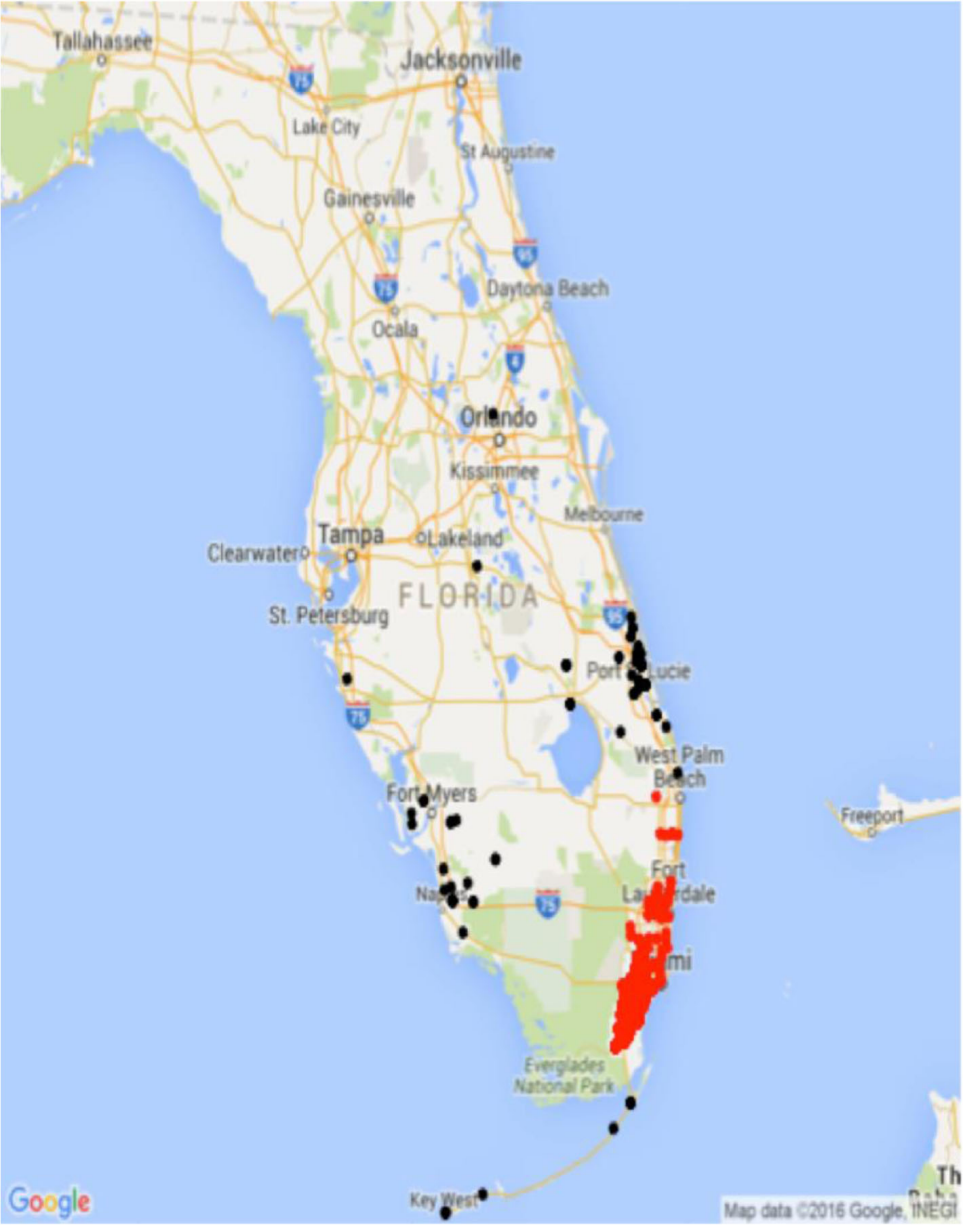
Injury locations for the two main clusters of patients cared for at the TC

We examined the K-means clustering solution using both statistics (minimized within group sum of squares) and interpretability. We found that a two-cluster solution was most parsimonious. The two-cluster solution accounted for 91.9% of the variation of the injuries arriving to our TC. The GIS map in Fig. 1 plots the injury types by location in Miami Dade county. Figure 2 plots the injury locations for the two main clusters who were cared for at our TC.

Table 2 displays the distribution of injuries referred to our institution based on the cluster of origin. Using chi-square test of independence, we found a significant relationship between the cluster of origin and the injury etiology. Motor vehicle injuries were responsible for 38.7% of the injured patients originating from the local Miami-Dade-Broward cluster. The majority of patients (96.3%) who came from outside the local cluster, suffered “Other” injuries (burns, bites) and were White, Other race, and non-Hispanic, *p* < 0.001. There was also a significant difference between the clusters based on age, as the patients arriving from Miami-Dade cluster were older (16–21 years old), *p* < 0.001 and those arriving from outside Miami-Dade were younger (mean age 6.8 years, *p* < 0.001).

**Table 2:** Distribution of Injuries by Cluster

	Miami-Dade-Broward	Outside Miami-Dade-Broward
Fall	103 (9.4%)	0 (0.0%)
Gunshot Wound	98 (8.9%)	0 (0.0%)
Motor Vehicle Injuries	425 (38.7%)	2 (2.5%)
Motorcycle Injuries	105 (9.6%)	1 (1.3%)
Other (burns, bites)	280 (25.5%)	77 (96.3%)
Pedestrian Injuries	46 (4.2%)	0 (0.0%)
Stabbing	42 (3.8%)	0 (0.0%)

Prior to the modeling process, we recoded the Race variable into Black (20.3%), Hispanic (47.8%), White (27.9%) and Other or Unknown (3.7%). The logistic regression model, using a penalized likelihood ratio test, was then performed to ascertain the effects of age, race, ISS, TRISS, ICU days and hospital length of stay on the likelihood that patients would survive their injuries. These results were not statistically significant, *p* = 0.90.

**Fig. 3:**
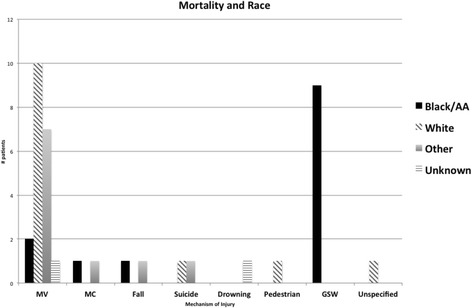
Association of mortality and race

A multinomial regression model was performed to ascertain the effects of age, race and ethnicity on the likelihood of suffering specific injuries, such as gunshot wounds, motor vehicle, motorcycle, pedestrian, stabbing or other injuries. The reference group was Black (Table 3). This model was significant, ×2 (42) = 278.53, *p* < 0.0001. The model explained 40.2% (Nagelkerke R^2^) of the variance. Hispanics had higher relative risks of falls (RR 10.4, 95% CI 2.7-29); higher relative risks for motor vehicle injuries (RR 6.4, 95% CI 3.6–11.4) and higher relative risks for motorcycle injuries (RR 3.7, 95% CI 1.7–8.2) than Black/AA children. Whites also had higher relative risks of falls (RR 6.42, 95% CI 2.5–16.5) than Black/AA children. Overall, Black/AA children had higher risks of gunshot wounds and of resultant mortality (Fig. 3). Interestingly, all mortalities for gunshot wounds in Black/AA children occurred within the same longitude and latitude areas of their residence. No significant differences were observed when adjusting for age and race for stabbing and pedestrian injuries.

**Table 3:** Multinomial Regression Analysis of Injury Type and Race/Ethnicity

		Risk	RRR	Lower 95% CI	Upper 95% CI	*P*-Value
Fall	Black	0.02	1.00			
	Hispanic or Latino	0.13	10.40	3.73	29.03	0.000
	Other	0.09	3.95	0.83	18.70	0.083
	White	0.14	6.42	2.50	16.48	0.000
Gunshot	Black	0.28	1.00			
	Hispanic or Latino	0.05	0.37	0.18	0.79	0.010
	Other	0.03	0.12	0.01	0.91	0.041
	White	0.02	0.08	0.04	0.16	0.000
Motor Vehicle	Black	0.13	1.00			
	Hispanic or Latino	0.44	6.42	3.62	11.38	0.000
	Other	0.35	2.93	1.18	7.28	0.021
	White	0.33	2.91	1.80	4.71	0.000
Motorcycle	Black	0.06	1.00			
	Hispanic or Latino	0.11	3.69	1.67	8.16	0.001
	Other	0.09	1.65	0.40	6.70	0.487
	White	0.10	1.94	0.98	3.81	0.056
Pedestrian	Black	0.06	1.00			
	Hispanic or Latino	0.02	0.74	0.25	2.22	0.593
	Other	0.03	0.51	0.06	4.23	0.530
	White	0.03	0.52	0.24	1.13	0.099
Stabbing	Black	0.06	1.00			
	Hispanic or Latino	0.03	0.96	0.34	2.75	0.945
	Other	0.06	1.10	0.22	5.52	0.910
	White	0.05	0.95	0.46	1.97	0.892

## Discussion

Our results indicate that there are racial, ethnic and gender differences in the pattern of injuries and their outcomes in the pediatric population attended at the Regional Trauma Center in South Florida. To the best of our knowledge, this is the first report of disparities in injury patterns and outcomes in the pediatric population in South Florida. The analysis demonstrated a troubling number of children and teens succumbing to gunshot wounds and motor vehicle injuries, with evident ethnic and racial variations as demonstrated by the multinomial analysis. Males are known to be more susceptible for unintentional injuries and our results also confirm this pattern (Sorenson 2011). Furthermore, we observed that most of the injuries occurred in the 16–21 years group, consistent with the standard referral patterns to a level 2 Trauma Center. Our institution is also a Burn Center, which explains the referral patterns of young children transferred from outside the Miami-Dade-Broward cluster to our institution.

The geographic distribution maps allowed us to identify areas in the various zones that have higher incidence of specific injuries in our county. As we transition to a Level 1 TC, we suspect that the injury referral patterns will change.

The disparities observed in this study are concordant to what is reported in the literature. Studies in adult populations have found that both race and insurance status are two independent predictors for mortality after trauma (Haider et al. 2008). In pediatric populations, this has also been confirmed. African American children have “over seven times more likely the risk of sustaining a burn or gunshot wound” and “3.5 times increased risk of death from preventable injury” (Brown 2010). Hispanics have been shown to have worse outcomes in both insured and uninsured groups. (Bernard et al. 2007; Fallat et al. 2006; Haider et al. 2008) Our study results that Hispanics were at higher risks of suffering falls, motor vehicle and motorbike injuries, but overall mortality rate was lower than for Whites and Black/AA.

One of our limitations of this retrospective study is that we did not account for insurance status for our patients. Additionally, we are only including data from patients seen at our Level 2 Trauma Center during the 5-year period, and did not include data from the pediatric trauma center, caring for children <16 years. Additional limitations include measurement and selection biases, the potential for misclassifications and inability to determine causality.

Efforts in planning injury prevention programs through passive interventions have been successful as proven by the development of safer highways, seatbelts, air bags, and playground designs (Gielen AC et al. 2006). However, prevention program developers still find it still challenging to modify the behavior of those who are at risk for injury, or of those who influence other’s behaviors and their environments (Gielen AC et al. 2006, Green and Kreuter 2005).

The current state of violence in the Miami-Dade County involving disproportionately deadly shootings in Black/AA teens have prompted a reaction from the local schools and authorities. In the last decade, more than 316 children and teens have been killed in Miami-Dade County due to gunfire violence (Rabin 2016). Parents complain about poor distribution of funds into their schools and the lack of presence of policy makers: “politicians came until June or July, then slowly stopped coming” (Rabin 2016). Student projects such as “Done with guns” are on the rise, and ideas are booming among young teens to create better solutions for prevention (Veiga 2016). These include better witness protection laws, improved afterschool programs for students, and social media educational campaigns to reach students wherever they are.

We recognize that there will be additional challenges in the development of injury prevention activities such as limited financial resources, time to develop and evaluate interventions, human resources to coordinate the programs and political opposition. Funding for gun violence prevention research has decreased and only 1/3 of 1% of publications on pediatric deaths are actually directed for gun violence research. (Violano et al. 2016, Stark and Shah 2017, Mack 2017).

**Fig. 4:**
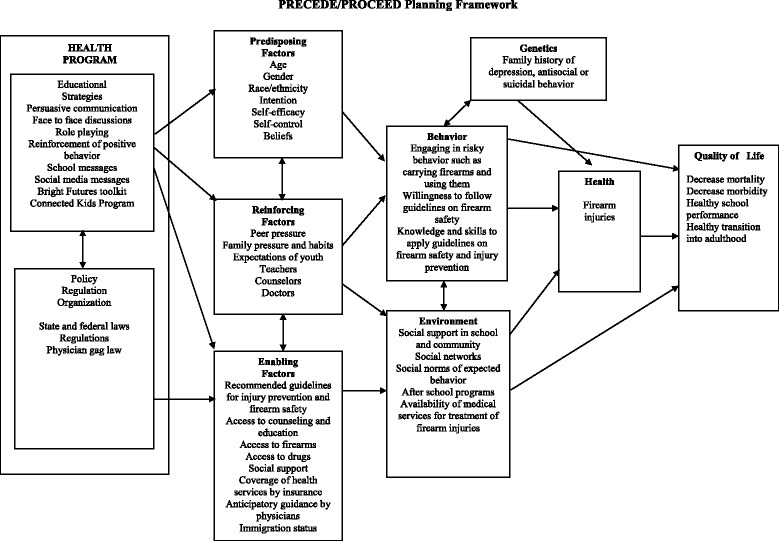
Precede-proceed framework for gun violence injury prevention

Our next phase for a prevention program development will be to use the Precede planning framework as a model for selecting and developing interventions, focusing on this vulnerable group of young victims of gun violence (Fig. 4) (Green and Kreuter 2005). Our cross-sectional analysis has provided us with some basic information that will allow us to target specific predisposing, reinforcing and enabling factors that are contributing to the patterns of injury and disparities. Critical evaluation of our interventions, with the collaboration of State and local coalitions, will be of utmost importance for the sustainability of the program in the community.

## Conclusion

There are racial, ethnic, and gender disparities in the patterns of injury and their outcomes in South Florida’s children. Efforts must be directed toward identifying risk factors and working in a standardized fashion with State and local coalitions using a well-planned framework.

## Abbreviations

AA: African American
CI: Confidence intervals
GCS: Glasgow coma scale
HLOS: Hospital length of stay
ICU: Intensive Care Unit
ISS: Injury severity scores
TC: Trauma center
TRISS: Trauma related injury severity scores
